# SAFE PHARMACOLOGIC APPROACHES TO PAIN MANAGEMENT IN GERIATRIC PATIENTS

**DOI:** 10.1093/geroni/igz038.1491

**Published:** 2019-11-08

**Authors:** Tatyana Gurvich

**Affiliations:** University of Southern California, Los Angeles, California, United States

## Abstract

We are in the midst of an opioid crisis, but have more patients in pain than ever before. Seniors have painful conditions such as chronic back pain, arthritis, neuropathy and fibromyalgia. As many as 50% of older Americans have pain daily from more advanced disease. CDC guidelines mandate that we use opioids less and adjuvant medications more. But using adjuvants in this patient population adds an extra layer of complexity. Some are not well tolerated contributing to cognitive decline, some have to be dose adjusted for declining kidney or liver function. Some medications are safe but underutilized by this patient population. Strategies for safe opioid use will be discussed from both provider and patient perspectives. A review of adjuvant medication will be provided focusing on safe medication use in seniors. Prescription and over-the-counter and alternative medications will be reviewed.

